# Safety of long-term use of potassium-competitive acid blockers: a narrative review

**DOI:** 10.3389/fphar.2026.1964932

**Published:** 2026-10-28

**Authors:** Yuqi Bao, Junkang Chen, Jiayu Han, Jingying Le, Haibiao Bao, Dejian Li

**Affiliations:** 1 The First Affiliated Hospital of Zhejiang Chinese Medical University (Zhejiang Provincial Hospital of Chinese Medicine), Hangzhou, China; 2 Pujiang County Hospital of Traditional Chinese Medicine, Jinhua, Zhejiang, China; 3 The Second Clinical Medical College of Zhejiang Chinese Medical University, Hangzhou, China

**Keywords:** drug-drug interactions, gastric mucosal effects, hypergastrinemia, long-term safety, potassium-competitive acid blockers, vonoprazan

## Abstract

**Introduction:**

Potassium-competitive acid blockers (P-CABs) reversibly inhibit gastric H^+^/K^+^-ATPase, providing rapid and potent acid suppression. They are increasingly used for gastroesophageal reflux disease, peptic ulcer disease, and *Helicobacter pylori* eradication. As maintenance therapy expands, long-term safety has drawn growing attention.

**Objective:**

To evaluate the available evidence on the long-term safety of P-CABs, distinguishing established findings from potential safety signals.

**Methods:**

This narrative review drew on searches of PubMed, Embase, Cochrane Library, and ClinicalTrials.gov through 30 June 2026. Studies with at least 24 weeks of treatment or follow-up formed the longer-term human evidence base. Evidence was synthesized by safety outcome, considering the specific agent and study design.

**Conclusion:**

Available comparative evidence, predominantly for vonoprazan, has not shown a substantially higher overall adverse event incidence than with proton pump inhibitors (PPIs). Sustained acid suppression can induce hypergastrinemia, which may be accompanied by parietal-cell hyperplasia, fundic gland dilatation, and other endoscopic mucosal changes. Evidence remains insufficient to determine the risks of rare or long-latency outcomes or to generalize long-term findings to all P-CABs. Long-term prescribing should include periodic reassessment of the indication and individualized monitoring.

## 1 Introduction

Potassium-competitive acid blockers (P-CABs) include vonoprazan, tegoprazan, keverprazan, fexuprazan, and zastaprazan. These agents competitively bind to the gastric H^+^/K^+^-ATPase at the potassium-binding site and reversibly inhibit gastric acid secretion. Their pharmacologic characteristics include a rapid onset of action, potent and sustained acid suppression, and relatively limited influence of the cytochrome P450 2C19 (CYP2C19) metabolizer phenotype (Mori and Suzuki, 2019). On the basis of these properties, several guidelines and clinical practice updates have included P-CABs as treatment options for selected acid-related disorders, including peptic ulcer bleeding (Gralnek et al., 2026), reflux esophagitis (Iwakiri et al., 2022; Patel et al., 2024), refractory acid reflux (Savarino et al., 2025), and *Helicobacter pylori (H. pylori)* eradication therapy.

Previous studies have shown that long-term use of proton pump inhibitors (PPIs) may be associated with hypergastrinemia and a range of histologic and endoscopic phenotypic changes (Kim, 2021). Because the acid-suppressive potency of P-CABs is generally at least comparable to that of PPIs, whether long-term P-CAB use aggravates these mucosal changes or further affects the risks of gastric cancer (GC) and gastric neuroendocrine tumors (gNETs) has become a central issue in long-term safety research (Aronson, 2016; Chen et al., 2023; Arai et al., 2024; Nagao et al., 2024).

Exposure to P-CABs in *H. pylori* eradication regimens generally lasts only 7–14 days; therefore, the resulting safety outcomes primarily reflect short-term tolerability and cannot be directly extrapolated to long-term maintenance therapy. Available comparative studies suggest that the overall safety of P-CABs is broadly similar to that of PPIs, with diarrhea, nausea, and abdominal discomfort being the most common adverse events, which are generally mild gastrointestinal symptoms (Dong et al., 2023; Du et al., 2024; Kong et al., 2024; Park et al., 2026). Long-term exposure occurs mainly in patients with gastroesophageal reflux disease (GERD) requiring maintenance therapy and in those at risk of recurrence or bleeding who require prolonged gastric mucosal protection. Accordingly, long-term safety assessments should focus on changes in gastric secretory function, gastric mucosal histology, tumor-related outcomes, and extra-gastric adverse events associated with sustained acid suppression.

Long-term human evidence for P-CABs remains limited and is concentrated predominantly on vonoprazan. Substantial differences among studies in population characteristics, indications, treatment doses, exposure duration, prior PPI exposure, *H. pylori* status, and outcome definitions limit cross-study comparisons and class-wide inferences. This narrative review aims to evaluate the available evidence on the long-term safety of P-CABs, focusing on gastric secretory function, gastric mucosal morphology and histologic changes, GC and gNET risk, and extra-gastric adverse events.

## 2 Literature search and narrative synthesis

This narrative review aimed to evaluate the available evidence on the long-term safety of P-CABs. PubMed, Embase, Cochrane Library, and ClinicalTrials.gov were searched from their inception to 30 June 2026. The search strategy combined terms for the drug class and individual agents (“potassium-competitive acid blocker,” “P-CAB,” “vonoprazan,” “tegoprazan,” “fexuprazan,” “zastaprazan,” and “keverprazan”) with terms related to long-term treatment and safety (“long-term,” “maintenance,” “extension,” “safety,” “tolerability,” “adverse event,” “hypergastrinemia,” “gastrin,” “gastric mucosa,” “histology,” “polyp,” “neoplasm,” “cancer,” “neuroendocrine tumor,” “microbiome,” “infection,” “hypomagnesemia,” “liver injury,” “kidney injury,” and “drug interaction”). Evidence considered in this review included interventional studies (including randomized trials and maintenance or extension studies), observational studies (including cohort and case–control studies), postmarketing surveillance studies, pharmacovigilance analyses, and case reports published in English that reported safety outcomes for P-CABs. The reference lists of included studies and relevant reviews were also screened manually. One author (YB) screened the retrieved records and selected relevant articles, and a second author (JC) checked the selection. Any disagreements were resolved through discussion. The final references were selected according to their relevance to the safety outcomes addressed in this review, with priority given to longer-term human evidence. In this review, studies with a treatment or follow-up duration of at least 24 weeks were defined as long-term human evidence (Table 1) (Ashida et al., 2016; 2018; Kawai et al., 2018; Mizokami et al., 2018; Cho et al., 2023; Laine et al., 2023; 2024; Xiao et al., 2024; Hong et al., 2025; Kim et al., 2026). This pragmatic cutoff was chosen to capture maintenance and extension studies. The observation period required to assess safety depends on the outcome, and substantially longer follow-up is needed to evaluate rare or long-latency outcomes such as gastric cancer and gastric neuroendocrine tumors. Short-term clinical studies, animal experiments, pharmacovigilance analyses, and case reports were used only as supplementary evidence for mechanistic interpretation or safety-signal detection.

**TABLE 1:** Randomized controlled trials and extension studies of P-CAB therapy with study durations of ≥24 weeks.

Country/year	Study design and population	Interventions and comparators	Sample size	Treatment duration	Main safety findings	References
Japan, 2016	Multicenter, randomized, double-blind, parallel-group, noninferiority healing trial; patients with endoscopically confirmed EE who achieved healing were rerandomized to a single-blind long-term maintenance study	Vonoprazan 10 or 20 mg once daily; no PPI comparator during maintenance	Long-term maintenance, n = 305: 154/151	52 weeks	TEAEs: 76.6% and 78.8%; SAEs: 5.2% and 7.3%. Nasopharyngitis was the most common AE (21.4% and 28.5%). Serum gastrin increased dose-dependently and declined after treatment discontinuation. Overall tolerability was favorable, with no new safety signals	(Ashida et al., 2016)
Japan, 2018	Phase III, multicenter, randomized, double-blind, active-controlled noninferiority trial followed by a single-blind, parallel-group extension; patients receiving long-term NSAIDs and at risk of recurrent peptic ulcer	Vonoprazan 10 or 20 mg; lansoprazole 15 mg once daily	Randomized, n = 642; safety set, n = 640: 218/212/210; extension, n = 406	Double-blind phase, 24 weeks; extension up to 104 weeks	TEAEs: 84.4%, 82.5%, and 88.1%; SAEs: 8.3%, 14.2%, and 8.6%. Discontinuation due to AEs was more frequent in the 20-mg group, but overall safety was comparable with lansoprazole. Serum gastrin increased dose-dependently with vonoprazan; no new long-term safety concerns were identified	(Mizokami et al., 2018)
Japan, 2018	Multicenter, randomized, double-blind noninferiority trial followed by a single-blind extension; patients with a history of peptic ulcer who required long-term LDA therapy	Vonoprazan 10 or 20 mg; lansoprazole 15 mg once daily	Randomized, n = 621: 202/202/217; extension, n = 439	Double-blind phase, 24 weeks; follow-up up to 98 weeks	TEAEs: 87.6%, 87.1%, and 84.8%; SAEs: 16.3%, 15.8%, and 14.7%, with broadly similar rates across groups. Serum gastrin increased with vonoprazan, but no patient discontinued because of hypergastrinemia. Gastrointestinal malignancies were reported in 3, 6, and 1 patients, respectively; two cases in the 20-mg group were present before dosing	(Kawai et al., 2018)
Japan, 2018	Phase III, multicenter, randomized, double-blind, active-controlled trial; patients with endoscopically healed EE after initial vonoprazan therapy	Vonoprazan 10 or 20 mg; lansoprazole 15 mg once daily	n = 607: 202/204/201	24 weeks	TEAEs: 54.0%, 58.8%, and 51.2%; serious TEAEs: 2.5%, 2.0%, and 2.0%. One case each of atrial fibrillation and abnormal liver function in the 20-mg group was considered possibly treatment-related. Serum gastrin and pepsinogen I/II increased dose-dependently; gastric mucosal histology showed no clinically meaningful changes	(Ashida et al., 2018)
United States and Europe, 2023	Phase III, multicenter, randomized, double-blind trial; patients with healed EE after up to 8 weeks of initial therapy entered the maintenance phase	Vonoprazan 10 or 20 mg; lansoprazole 15 mg once daily	Maintenance safety set, n = 889: 296/296/297	24 weeks	TEAEs: 54.1%, 56.4%, and 50.5%; SAEs: 3.4%, 4.7%, and 2.4%. COVID-19 was the most common AE (6.1%, 10.1%, and 6.7%). Fractures occurred in 0.7%, 1.4%, and 0.3%, and no CDI occurred. Serum gastrin was higher with vonoprazan; gastric biopsies showed no clinically meaningful neoplastic changes, and changes in neuroendocrine-cell proliferation markers were limited overall	(Laine et al., 2023)
South Korea, 2023/2025	Phase III, multicenter, randomized, double-blind trial in patients with healed mild EE after initial therapy; the 2025 publication was a secondary analysis of serum gastrin from the same trial	Tegoprazan 25 mg; lansoprazole 15 mg once daily	Randomized, n = 351; safety set, n = 347: 173/174	24 weeks	TEAEs: 28.3% and 37.9%; drug-related TEAEs: 12.7% and 21.3%. Two SAEs in the tegoprazan group were unrelated to treatment, and no patient discontinued tegoprazan because of a TEAE. In the secondary analysis, serum gastrin levels and the proportion of patients with hypergastrinemia at weeks 16 and 24 were lower with tegoprazan than with lansoprazole	(Cho et al., 2023; Lee et al., 2025a)
China, South Korea, and Malaysia, 2024	Phase III, multicenter, randomized, double-blind, double-dummy trial; patients with healed EE after initial therapy	Vonoprazan 10 or 20 mg; lansoprazole 15 mg once daily	Safety set, n = 703: 235/226/242	24 weeks	TEAEs: 66.8%, 69.0%, and 65.3%; upper respiratory tract infection: 12.8%, 12.8%, and 8.7%. SAEs occurred in 4.3%, 3.5%, and 5.0%, with no treatment-related SAEs. Serum gastrin increased dose-dependently in the vonoprazan groups; ALT elevations were similar across groups (5.5%, 4.0%, and 6.6%)	(Xiao et al., 2024)
United States, 2024	Multicenter, randomized, double-blind, placebo-controlled trial followed by a 20-week active-treatment extension; adults with frequent heartburn and no EE on endoscopy	Vonoprazan 10 or 20 mg once daily; placebo during the first 4 weeks, followed by vonoprazan for all participants	Initial phase, n = 772; extension safety set, n = 723	24-week study; vonoprazan exposure, 20–24 weeks	During the extension, AEs occurred in approximately 31%–34% across groups; COVID-19 was the most common AE (10.2% and 8.5%). SAEs occurred in 1.3%–3.3% and were considered unrelated to treatment. Fractures were rare and no CDI occurred. Serum gastrin increased during treatment, more prominently with 20 mg, and approached baseline 4 weeks after discontinuation	(Laine et al., 2024)
Japan, 2023/2025 (same VISION cohort)	Phase IV, multicenter, randomized, open-label trial; H. pylori-negative patients with healed EE, with emphasis on gastric mucosal histologic safety	Healing: vonoprazan 20 mg or lansoprazole 30 mg; maintenance: 10 mg or 15 mg once daily, with protocol-specified dose escalation	Randomized, n = 208: 139/69; maintenance safety set, n = 202: 135/67	260 weeks	The prespecified gastric mucosal histopathology assessment showed no malignant epithelial changes; no gastric NETs were observed over 5 years; one gastric adenoma occurred in each group. Serum gastrin, parietal-cell hyperplasia, and foveolar hyperplasia were more pronounced with vonoprazan, whereas ECL-cell hyperplasia was not higher than with lansoprazole. AE and SAE profiles were generally similar	(Haruma et al., 2023; Uemura et al., 2025)
South Korea, 2025	Phase III, multicenter, randomized, double-blind, active-controlled trial; patients with musculoskeletal disorders requiring long-term NSAID therapy and having risk factors for peptic ulcer	Fexuprazan 20 mg; lansoprazole 15 mg once daily	Randomized, n = 423; safety set, n = 419: 210/209	24 weeks	TEAEs: 33.81% and 39.23%; ADRs: 8.57% and 4.78%; the most common ADRs were gastrointestinal. SAEs occurred in 3.81% and 1.91%. One fracture was reported in each group. CDI was not reported. Changes in serum gastrin did not differ significantly between groups, and overall safety was comparable	(Hong et al., 2025)
South Korea, 2026	Phase III, multicenter, randomized, double-blind, active-controlled trial; patients requiring continuous NSAID therapy	Tegoprazan 25 mg; lansoprazole 15 mg once daily	Randomized, n = 392; safety set, n = 386: 191/195	24 weeks	TEAEs: 28.80% and 34.36%; SAEs: 1.57% and 2.56%; ADRs: 13.09% and 16.41%. One serious ADR occurred in each group (0.52% and 0.51%). Laboratory safety findings were generally comparable; serum gastrin was not reported	(Kim et al., 2026)

Percentages are presented in the order of the treatment groups listed in the “Interventions and comparators” column. Sample sizes preferentially refer to the safety analysis set for the long-term phase. Because indications, follow-up durations, and definitions of AEs, differed across studies, AE/TEAE, incidences should not be compared directly between studies.

**Abbreviations:** ADR, adverse drug reaction; AE, adverse event; ALT, alanine aminotransferase; CDI, *Clostridioides difficile infection*; COVID-19, coronavirus disease 2019; ECL, enterochromaffin-like cell; EE, erosive esophagitis; *H. pylori*, *Helicobacter pylori*; LDA, low-dose aspirin; NET, neuroendocrine tumor; NSAID, nonsteroidal anti-inflammatory drug; P-CAB, potassium-competitive acid blocker; PPI, proton pump inhibitor; SAE, serious adverse event; TEAE, treatment-emergent adverse event.

Because the included studies showed substantial heterogeneity in the specific drug evaluated, study population, indication, exposure duration, and outcome definitions, no meta-analysis was performed. Evidence was synthesized narratively by safety outcome. No formal risk-of-bias assessment was performed. Evidence was interpreted with particular consideration of study design, sample size, exposure duration, outcome type, and capacity for causal inference. Randomized controlled trials and extension studies were used mainly to evaluate relatively common long-term adverse events and histologic changes; observational studies and pharmacovigilance data were used mainly to identify rare or long-latency signals; and case reports were used only to raise alerts rather than to establish causality.

## 3 Hypergastrinemia and gastric mucosal changes

### 3.1 Gastrin response and histologic changes

Sustained inhibition of the H^+^/K^+^-ATPase by P-CABs increases intragastric pH and reduces acid stimulation of antral D cells, thereby decreasing somatostatin secretion, relieving paracrine inhibition of G cells, and promoting gastrin release. Circulating gastrin primarily stimulates histamine synthesis and release through cholecystokinin-2 (CCK_2_) receptors on enterochromaffin-like (ECL) cells; histamine subsequently acts on histamine H_2_ receptors on parietal cells, generating a compensatory acid-stimulatory signal (Engevik et al., 2020). Gastrin can also act directly on CCK_2_ receptors on parietal cells, but when the H^+^/K^+^-ATPase remains inhibited, these compensatory pathways cannot restore normal gastric acid secretion (Figure 1). Thus, hypochlorhydria may coexist with hypergastrinemia and, under prolonged stimulation, induce adaptive remodeling of the gastric glands. Hypergastrinemia has therefore become an important intermediate biological phenotype in the safety assessment of long-acting acid suppression.

**FIGURE 1:**
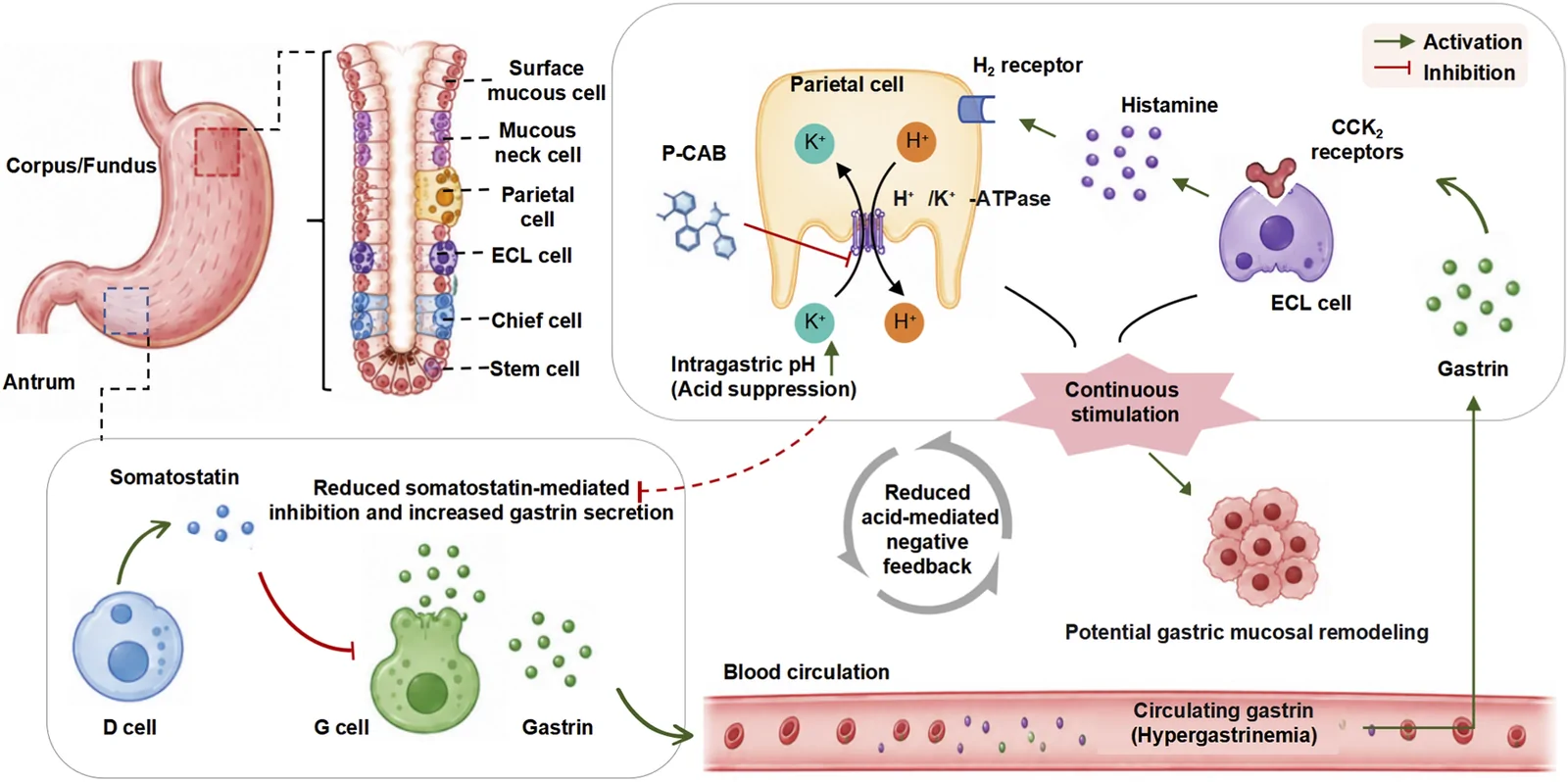
Proposed mechanisms linking sustained P-CAB-mediated acid suppression to hypergastrinemia and gastric mucosal remodeling. Abbreviations: CCK_2_, cholecystokinin-2 receptor; ECL, enterochromaffin-like cell; H_2_ receptors, histamine H_2_ receptor; P-CAB, potassium-competitive acid blocker.

Available studies consistently show that gastrin levels increase during P-CAB therapy, although the magnitude of this increase is influenced by the specific agent, dose, *H. pylori* status, and degree of gastric mucosal atrophy. In patients with PPI-refractory reflux esophagitis who received P-CAB maintenance therapy for 96 weeks, serum gastrin remained elevated, but no carcinoid-related events were reported during the study (Mizuno et al., 2025). A meta-analysis showed a greater increase in gastrin levels with P-CABs than with PPIs, with a pooled mean difference of 130.92 pg/mL (95% CI, 36.37–225.47); however, the included studies were heterogeneous with respect to the drug, dose, measurement time point, and baseline gastric mucosal status (Jang et al., 2026). In contrast, in a 24-week maintenance study, both serum gastrin levels and the proportion of patients with hypergastrinemia at weeks 16 and 24 were lower with tegoprazan 25 mg than with lansoprazole 15 mg; age, sex, baseline gastrin level, *H. pylori* status, and treatment assignment were each independently associated with the gastrin level at the end of treatment (Lee K. J. et al., 2025). These findings suggest that gastrin responses may differ among P-CAB regimens, although the available data are insufficient to establish a robust agent-specific ranking.

A study of 48 patients who received vonoprazan 10 mg continuously for more than 1 year showed that gastrin levels increased markedly early in treatment and reached a plateau after approximately 1.5 years; severe gastric mucosal atrophy was independently associated with higher gastrin levels (Shinozaki et al., 2022a). Another prospective cross-sectional study found that, among patients with mild or no atrophic gastritis, gastrin levels were higher in vonoprazan users than in conventional PPI users, whereas this difference was no longer evident among patients with severe atrophic gastritis (Kojima et al., 2018). These findings indicate that baseline gastric mucosal status can substantially modify the drug-associated gastrin response.

The magnitude of hypergastrinemia varies across P-CAB regimens and is influenced by dose, pharmacokinetic properties, baseline gastric mucosal atrophy, and *H. pylori* status. Serum gastrin concentrations should therefore be interpreted in the context of the specific treatment regimen and the patient’s gastric mucosal background rather than as a uniform class-level response. Long-term potent acid suppression may also be accompanied by persistent histologic adaptation. In the VISION study, the proportions of patients with parietal-cell hyperplasia and foveolar epithelial hyperplasia at week 260 were higher in the vonoprazan group than in the lansoprazole group, whereas the proportions with ECL-cell and G-cell hyperplasia were similar. No gNETs were observed in either group, and the prespecified gastric mucosal histopathology assessment showed no malignant epithelial changes (Uemura et al., 2025). Separately reported gastric neoplastic events are discussed in Section 4. In VISION, vonoprazan was associated with more pronounced parietal-cell and foveolar epithelial hyperplasia than lansoprazole, whereas ECL-cell hyperplasia was similar between groups. These histologic differences were not accompanied by malignant epithelial changes during the 5-year study period. Parietal-cell loss, chief-cell alterations, and metaplasia-like responses observed in animal studies may support mechanistic hypotheses, but differences in exposure multiples and tissue responses preclude direct extrapolation to humans (Edelmuth et al., 2023).

### 3.2 Endoscopic mucosal phenotypes

Gastric mucosal redness was among the earliest endoscopic phenotypes associated with P-CAB use to attract attention. Case series have shown that the lesions are commonly located along the greater curvature of the gastric body or fundus and appear as linear or patchy areas of redness. Histologically, they are characterized by fundic gland dilatation, parietal-cell protrusion and vacuolar degeneration, with vascular congestion, focal hemorrhage, or inflammatory-cell infiltration (Kubo et al., 2020; 2021). In a retrospective study of 1,101 patients, the detection rates in the control, H_2_-receptor antagonist (H_2_RA), PPI, and vonoprazan groups were 1.9%, 1.5%, 6.2%, and 8.7%, respectively. Both PPI and vonoprazan use were independently associated with this finding, but no clear gradient according to treatment duration was observed (Shinozaki et al., 2022b). The occurrence of gastric mucosal redness in both PPI and vonoprazan users is more consistent with a response to sustained acid suppression than with a lesion specific to vonoprazan.

Yoshizaki et al. described stardust gastric mucosa associated with vonoprazan use. It appears as small, nonremovable white elevations, predominantly in the upper third of the stomach. Histologically, it consists of mucus accumulation within dilated glandular ducts surrounded by flattened glandular epithelium. After propensity score matching, the detection rate was higher among vonoprazan users than among nonusers (4.9% vs. 0.2%), and the cumulative incidence increased over time to 4.6%, 16.5%, and 26.2% at 1, 2, and 3 years, respectively; treatment for more than 205 days and female sex were independently associated factors (Yoshizaki et al., 2021). Stardust gastric mucosa and cobblestone-like changes appear to become more frequent with longer exposure, although their clinical significance remains unclear because bleeding, dysplasia, and tumor progression have not been demonstrated (Haruma et al., 2023).

Another endoscopic change associated with long-term acid suppression is the white-spot appearance. One study found that white spots were detected more frequently in users of acid-suppressive agents than in nonusers and were particularly common in patients with hypergastrinemia and in P-CAB users, suggesting a possible association with potent acid suppression and elevated gastrin levels (Nishiyama et al., 2021). Case reports have also described multiple white globular lesions after vonoprazan use; some lesions decreased in size or disappeared after switching to a PPI, although parietal-cell protrusion and glandular dilatation persisted (Miwa et al., 2021a; 2021b). P-CAB exposure may therefore explain some nonneoplastic white lesions, but these findings still require endoscopic and pathologic differentiation from early GC and other lesions. Gastric black spots and other white phenotypes may also be influenced by *H. pylori* infection status, post-eradication mucosal repair, and prior PPI exposure (Hatano et al., 2018; Adachi et al., 2019) and should not be attributed exclusively to a single drug.

Web-like mucus is another recently described gastric phenotype, appearing endoscopically as spiderweb-like or reticular mucus adherent to the gastric mucosal surface. Among 547 patients undergoing endoscopy, the overall detection rate was approximately 6%, whereas the finding was present in approximately 19% of vonoprazan users; however, no significant trend between treatment duration and detection rate was identified (Shinozaki et al., 2025b). Available studies suggest an association between web-like mucus and vonoprazan exposure (Kaneko et al., 2024; Kubo et al., 2024), but its diagnostic specificity, time dependence, and clinical consequences remain uncertain.

Among these endoscopic findings, gastric mucosal redness and cobblestone-like changes are relatively well described, whereas stardust gastric mucosa and web-like mucus remain emerging findings supported by limited evidence.

### 3.3 Gastric polyps

Fundic gland polyps and gastric hyperplastic polyps are among the most readily visible mucosal changes associated with long-term acid suppression. Chronic hypochlorhydria and hypergastrinemia may promote increases in polyp number or size by altering glandular secretion, parietal-cell morphology, and trophic signaling within the mucosa (Suda et al., 2026). However, P-CAB-associated mucosal phenotypes do not invariably parallel gastrin levels; a single gastrin measurement neither predicts polyp progression nor should serve as the sole basis for discontinuing treatment (Shinozaki et al., 2025a).

Case reports suggest that some polypoid lesions may be reversible. A giant red gastric polyp gradually decreased in size at 10 and 22 months after vonoprazan discontinuation (Iwamuro et al., 2022). In three cases of severely bleeding gastric hyperplastic polyps reported in 2026, polyps that had not been resected regressed and anemia improved after vonoprazan was discontinued or replaced with an H_2_RA. Two of the patients were undergoing hemodialysis, and impaired gastrin clearance together with an increased bleeding tendency may have amplified the clinical consequences (Kasuga et al., 2026).

A hereditary high-risk background may further modify the polyp response. In a patient with familial adenomatous polyposis, the numbers of fundic gland polyps and colorectal adenomas increased during vonoprazan treatment and decreased after discontinuation, in parallel with changes in gastrin levels (Urabe et al., 2024). A duodenal polyp developed in a patient receiving long-term PPI/P-CAB therapy and disappeared 1.5 months after treatment withdrawal, suggesting that acid-suppression-related proliferative responses may not be confined to the stomach (Shintaku et al., 2021). A white change in a nonampullary duodenal adenoma during P-CAB therapy has also been reported, although the nature of the lesion and its causal relationship with the drug remain unclear (Matsuda et al., 2019). Because this evidence is derived mainly from case reports, it can be used only to identify potential safety signals and cannot establish incidence or causality.

## 4 Risk of GC and gNETs

Human evidence relating P-CAB exposure to tumor outcomes includes long-term randomized studies, observational database studies, and case reports, supplemented by animal carcinogenicity data for mechanistic interpretation. These evidence sources differ substantially in the specific drug, exposure dose and duration, host gastric mucosal background, and outcome definitions (Table 2) (Saito et al., 2021; Arai et al., 2023; 2024; Haruma et al., 2023; Nagao et al., 2024; Howden et al., 2025; Uemura et al., 2025; Zhong et al., 2025; Moriguchi et al., 2026; Shiratori et al., 2026). Interpretation therefore requires clear distinction among biological plausibility, safety signals, and clinical evidence of causality.

**TABLE 2:** Clinical evidence on P-CAB exposure and tumor-related outcomes in humans.

Country/year	P-CAB	Study design	Sample size and population	Exposure dose and duration	Tumor-related outcomes	References
Japan, 2021	Vonoprazan	Case report	A 51-year-old man with RE; H. pylori-negative, with no family history of gastric cancer	Induction with 20 mg/day for 4 weeks, followed by maintenance with 10 mg/day; the first lesion was detected at week 156 and the second at week 164	Two raspberry-like foveolar-type gastric adenocarcinomas were detected sequentially in the gastric body (approximately 5 and 4 mm); hypergastrinemia was present	(Saito et al., 2021)^b^
Japan, 2023	P-CAB (specific agent and daily dose not reported)	Multicenter retrospective cohort study (research letter)	A total of 2,338 patients underwent *H. pylori* eradication at nine hospitals between 2014 and 2019; 241 were P-CAB users	P-CAB exposure was defined as prescriptions for >60 days; individual cumulative dose and actual exposure duration were not further reported	Gastric cancer occurred in 12/241 P-CAB users (4.98%); adjusted HR, 2.54 (95% CI, 1.34–4.81). Using patients who received no H_2_RA, PPI, or P-CAB as the reference, the adjusted HR was 3.55 (95% CI, 1.69–7.46)	(Arai et al., 2023)^e^
Japan, 2023	Vonoprazan	Phase IV, multicenter, randomized, open-label, parallel-group trial (3-year interim analysis of VISION)	A total of 208 H. pylori-negative patients with EE were randomized (vonoprazan, n = 139; lansoprazole, n = 69); 202 entered maintenance, with safety sets of 135 and 67, respectively	Healing phase: 20 mg/day for 4–8 weeks; maintenance phase: starting dose 10 mg/day, with escalation to 20 mg/day if required; interim analysis through week 156	One patient in the vonoprazan group had an approximately 5-mm foveolar-type gastric adenoma detected at week 156, followed by a similar approximately 4-mm lesion 8 weeks later. Prespecified gastric mucosal biopsies showed no neoplastic or dysplastic changes, and no gastric NET was observed	(Haruma et al., 2023)^a,b^
Japan, 2024	Vonoprazan (after prior lansoprazole)	Case report	A 52-year-old man with GERD and prior *H. pylori* eradication; no family history of MEN1 or VHL.	Lansoprazole for 11 years, followed by vonoprazan for 3 years; the vonoprazan dose was not reported	Three G1 NETs in the gastric body (9, 5, and 5 mm), accompanied by hypergastrinemia, linear ECL-cell hyperplasia, and G-cell hyperplasia. The lesions were removed endoscopically; after potent acid suppression was discontinued, no recurrence or new lesions were observed during follow-up	(Nagao et al., 2024)^c^
Japan, 2024	Vonoprazan	Population-based retrospective cohort study using the DeSC Healthcare claims database; propensity-score-matched analysis	A total of 54,055 patients after *H. pylori* eradication; patients with eradication failure, previous gastric cancer, <1 year of follow-up, or gastric cancer diagnosed within 1 year after eradication were excluded. After matching, the P-CAB and H_2_RA groups each included 2,120 patients	Long-term use was defined as use of a single acid suppressant for >6 months and categorized as <1, 1–2, 2–3, or ≥3 years. High dose: 20 mg for >2 months; low dose: 10 mg, or 20 mg for ≤2 months	Gastric cancer occurred in 40/2,120 P-CAB users (1.89%) and 21/2,120 H_2_RA users (0.99%); HR, 1.92 (95% CI, 1.13–3.25). The HR was 2.36 for use ≥3 years and 3.01 for high-dose use; risk did not differ significantly from that with PPIs	(Arai et al., 2024)^e^
Japan, 2025	Vonoprazan	Phase IV, multicenter, randomized, open-label, parallel-group trial (5-year final analysis of VISION)	A total of 208 H. pylori-negative patients with EE were randomized; 202 entered maintenance (safety set: vonoprazan, n = 135; lansoprazole, n = 67)	Healing phase: 20 mg/day for up to 8 weeks; maintenance phase: 10 mg/day for 260 weeks, with escalation to 20 mg/day if control was inadequate	One foveolar-type gastric adenoma was reported in the vonoprazan group at week 156; one oxyntic gland adenoma was reported in the lansoprazole group at week 204. The prespecified gastric mucosal histopathology assessment showed no malignant epithelial changes; no gastric NETs were observed in either group	(Uemura et al., 2025)^a,b^
Multiple countries, 2025	Vonoprazan	Integrated safety analysis of 14 adult clinical trials plus postmarketing pharmacovigilance data	Clinical trials included 5,318 vonoprazan-treated patients, 2,011 PPI-treated patients, and 779 placebo recipients across GERD/EE populations in Asia, North America, and Europe	Main regimens were 10 or 20 mg once daily; some studies used 20 mg for healing followed by 10 or 20 mg for maintenance. Mean exposure was 142.1 days (2,068 person-years); postmarketing data were collected through 25 December 2023	One gastric malignancy was reported in each of the vonoprazan and lansoprazole groups; both patients were from VISION; the vonoprazan case also overlapped with the Saito case report. Postmarketing surveillance identified 69 serious gastric NET events with vonoprazan monotherapy and 5 with combination therapy; event counts do not necessarily represent verified, independent cases	(Howden et al., 2025)^b,d^
Global (FAERS), 2025	Vonoprazan	Pharmacovigilance and disproportionality analysis of spontaneous FAERS reports	From the first quarter of 2023 through the first quarter of 2025, 978 reports identified vonoprazan as the primary suspected drug	Case-level dose, cumulative exposure, and latency before tumor onset could not be reliably determined	Reports included 11 gastric cancers, 4 esophageal adenocarcinomas, and 3 NETs; esophageal adenocarcinoma and NET remained disproportionality signals after FDR correction. These findings represent signal detection and cannot be used to estimate incidence or establish causality	(Zhong et al., 2025)^d^
Japan, 2026	Vonoprazan	Retrospective cohort study across 15 institutions	A total of 7,172 patients with previous gastric cancer resected endoscopically or surgically and subsequent *H. pylori* eradication; 431 were P-CAB users. Median follow-up was 5.8 years	Chronic use was defined as continuous exposure for >3 months and categorized as <1, 1–3, or >3 years; the daily dose was not reported	A total of 342 metachronous gastric cancers occurred. P-CAB use was associated with a higher risk of MGC (adjusted HR, 3.89); adjusted HRs for <1, 1–3, and >3 years of use were 3.44, 3.95, and 9.36, respectively. All participants had previous gastric cancer and therefore a high baseline risk	(Shiratori et al., 2026)^e^
Japan, 2026	Vonoprazan (after prior PPI therapy)	Case report	A 52-year-old man with RE; *H. pylori* IgG, anti-parietal-cell antibodies, and anti-intrinsic-factor antibodies were negative; he reported alcohol use and heavy smoking	Vonoprazan 10 mg/day for 6 years; prior PPI therapy was reported, but total exposure to potent acid suppression was unclear	A single 5-mm G1 NET on the anterior wall of the gastric body, with parietal-cell protrusion and hypergastrinemia (170 pg/mL). The resected specimen showed no vascular invasion, and there was no evidence of metastasis	(Moriguchi et al., 2026)^c^

a, Haruma et al. (2023) and Uemura et al. (2025) represent the 3-year interim and 5-year final analyses, respectively, of the same VISION, trial (NCT02679508) and should not be treated as independent study populations. b, The foveolar-type gastric lesions described by Saito et al. (2021), Haruma et al. (2023), Uemura et al. (2025), and Howden et al. (2025) relate to the same VISION, participant and should not be counted as independent tumor cases. The lesions were described as foveolar-type adenomas in the VISION, reports and as foveolar-type adenocarcinomas in the case report and integrated safety analysis. c, These case reports involved prior or sequential PPI, exposure; therefore, the tumors cannot be attributed solely to vonoprazan. d, Postmarketing and FAERS, findings are based on spontaneous reports and may be affected by duplicate reporting, missing clinical or exposure information, and reporting bias; they support signal detection but cannot estimate incidence or establish causality. e, Observational cohort estimates may be affected by residual confounding and confounding by indication; study populations and exposure definitions differed, and the MGC, cohort included only patients with previous gastric cancer.

**Abbreviations:** CI, confidence interval; ECL, enterochromaffin-like cell; EE, erosive esophagitis; FAERS, US, food and drug administration adverse event reporting system; FDR, false discovery rate; G1, grade 1; GERD, gastroesophageal reflux disease; H_2_RA, histamine-2 receptor antagonist; *H. pylori*, *Helicobacter pylori*; HR, hazard ratio; IgG, immunoglobulin G; MEN1, multiple endocrine neoplasia type 1; MGC, metachronous gastric cancer; NET, neuroendocrine tumor; P-CAB, potassium-competitive acid blocker; PPI, proton pump inhibitor; RE, reflux esophagitis; VHL, von Hippel–Lindau disease; VISION, vonoprazan study in patients with erosive esophagitis to evaluate long-term safety.

Persistent hypergastrinemia can stimulate ECL-cell proliferation and is an important biological basis for type I and type II gNETs. Type I gNETs usually arise in the setting of autoimmune gastritis or severe atrophic gastritis, whereas type II gNETs are associated with gastrinoma or multiple endocrine neoplasia type 1 (MEN1); host baseline risk should therefore not be conflated with drug effects. An increased risk of gNETs through the gastrin–ECL axis is biologically plausible under potent acid suppression, but adequate human evidence is lacking. Case reports may identify signals after extremely prolonged exposure, although their capacity for causal inference is limited. One patient who had received lansoprazole for 11 years followed by vonoprazan for 3 years developed three grade 1 gNETs accompanied by hypergastrinemia, linear ECL-cell hyperplasia, and G-cell hyperplasia. After conventional Rindi types I–III had been excluded, the authors considered the lesions potentially related to long-term acid suppression; no recurrence was observed after treatment withdrawal and endoscopic therapy (Nagao et al., 2024). Another patient developed a solitary 5-mm grade 1 gNET after 6 years of vonoprazan 10 mg/day following prior PPI therapy (Moriguchi et al., 2026). Because these cases involved concomitant or sequential PPI exposure, lacked control groups, and provided limited information on baseline risk, they support only signal detection and cannot be attributed to vonoprazan monotherapy. In a long-term carcinogenicity study of tegoprazan, gastric ECL-cell tumors developed in rats under high-exposure conditions, mainly at exposures approximately sevenfold higher than the recommended human exposure (Kim et al., 2023). This finding supports mechanistic plausibility but cannot be directly extrapolated to human risk at routine clinical doses.

In the VISION study, patients with erosive esophagitis were randomized before the healing phase; those with confirmed healing subsequently entered maintenance therapy for up to 260 weeks, starting with vonoprazan 10 mg or lansoprazole 15 mg once daily. The prespecified gastric mucosal histopathology assessment showed no malignant epithelial changes, no gNETs were observed, and the rate of ECL-cell hyperplasia was not higher with vonoprazan than with lansoprazole. Separately, the final VISION report described one gastric adenoma in each treatment group (Uemura et al., 2025). These events were reported as gastric malignancies in the integrated safety analysis, indicating differences in diagnostic terminology across reports rather than additional independent cases (Howden et al., 2025). An interim analysis of the same study showed that some endoscopic gastric mucosal phenotypes increased with treatment duration, with no neoplastic or dysplastic changes observed in the prespecified gastric mucosal biopsies (Haruma et al., 2023). These findings provide some reassurance regarding safety within 5 years; however, the sample size, open-label design, and relatively homogeneous study population remain insufficient to exclude rare tumor outcomes or those with latency periods exceeding 5 years.

Several observational studies have reported statistical associations between P-CAB exposure and GC risk, although the study populations and exposure definitions varied considerably. In a 2023 multicenter retrospective cohort study, GC developed in 12 of 241 patients who had received P-CAB prescriptions for more than 60 days, with an adjusted hazard ratio (HR) of 2.54; however, the specific agents, cumulative doses, and actual treatment durations were insufficiently reported (Arai et al., 2023). In a 2024 propensity score–matched study using the DeSC database, long-term P-CAB therapy was compared with H_2_RA therapy after *H. pylori* eradication; the incidences of GC were 1.89% and 0.99%, respectively, with an HR of 1.92, and an association with exposure duration or dose was reported (Arai et al., 2024). Another study found that P-CAB use was associated with metachronous GC among high-risk patients who had undergone resection of a previous GC and completed *H. pylori* eradication (Shiratori et al., 2026). These associations should be interpreted in light of differences in baseline GC risk, *H. pylori* status, gastric atrophy, previous GC, and the frequency of endoscopic surveillance, which may introduce residual confounding and detection bias. In addition, cases of foveolar-type gastric adenoma and adenocarcinoma arising within fundic gland polyps have been reported in long-term PPI users, both in *H. pylori*-negative settings (Kanai et al., 2025; Miyaoka et al., 2025); however, evidence remains insufficient to establish these neoplastic lesions as P-CAB-specific outcomes.

Overall, current evidence does not demonstrate that P-CABs directly increase the risk of GC or gNETs in the general population with acid-related disorders. Nevertheless, very long-term potent acid suppression warrants caution in high-risk patients, including those with severe gastric mucosal atrophy, intestinal metaplasia, autoimmune gastritis, previous GC, or hereditary polyposis syndromes. Clinical management should emphasize rigorous reassessment of the indication and endoscopic surveillance based on the patient’s pre-existing GC risk rather than treating P-CABs as universally contraindicated.

## 5 Microbiota, infection, and intestinal safety

### 5.1 Microbiota alterations

Gastric acid is an important barrier limiting the entry of oropharyngeal and environmental microorganisms into the distal gastrointestinal tract. A sustained increase in intragastric pH can weaken this barrier, allowing oral bacteria to survive and colonize the gastrointestinal tract and thereby altering the gastrointestinal microbiota. A randomized controlled study showed that even 7 days of PPI therapy can alter both the gastric and intestinal microbiota (Zhu et al., 2024). Similar findings have been observed during P-CAB therapy: an 8-week clinical trial showed that vonoprazan treatment was accompanied by reduced fecal microbial diversity and an increased relative abundance of *Streptococcus* (Furune et al., 2024). Animal studies further support an effect of vonoprazan on the gastrointestinal microbiota; after 12 weeks of continuous exposure in rats, alpha diversity, beta diversity, and the relative abundance of multiple taxa were altered (Najah et al., 2023). The insulin-gastrin transgenic (INS-GAS) model also suggests a possible biological link among potent acid suppression, microbiota alterations, and gastric mucosal inflammation (Peng et al., 2024).

The microbiota effects of individual P-CABs may not be uniform. In a murine model of inflammatory bowel disease, tegoprazan did not induce the same dysbiosis as rabeprazole and instead improved epithelial barrier function and inflammation (Son et al., 2022). A model of nonsteroidal anti-inflammatory drug–induced small-intestinal injury likewise showed divergent effects of different acid suppressants on microbial composition and mucosal injury (Li et al., 2022). Microbiota responses may differ among individual P-CABs and according to host and disease context. Current studies are largely mechanistic and short to medium term, so the clinical consequences of these changes during prolonged treatment remain undefined.

### 5.2 *Clostridioides difficile* infection, small-intestinal bacterial overgrowth, and other infections

*Clostridioides difficile* infection (CDI) is an important healthcare-associated infection influenced by multiple factors, including antimicrobial exposure, advanced age, underlying disease, and impairment of the gastric acid barrier. Associations between acid-suppressive therapy and CDI have been inconsistent (Kim et al., 2025). Available meta-analyses and observational studies have not demonstrated a consistent additional risk of CDI with P-CABs compared with PPIs or H_2_RAs (Watanabe et al., 2021; Floria et al., 2025; Kim et al., 2025). Regarding recurrent CDI, a single-center retrospective cohort study found that P-CAB use at the initial CDI episode was associated with lower odds of recurrence within 8 weeks (adjusted OR 0.33, 95% CI 0.12–0.90), although only 44 patients were receiving P-CABs (Hirai and Hanai, 2026). Previous studies have suggested that the incidence of small-intestinal bacterial overgrowth (SIBO) may increase during PPI therapy (Lombardo et al., 2010); however, in a retrospective study of patients receiving acid suppression for at least 3 months, glucose breath test positivity did not differ significantly between tegoprazan and PPI users (22.8% vs. 31.7%; p = 0.15) (Lim et al., 2024). Overall, observational studies have not shown a consistent excess risk of CDI or SIBO with P-CABs compared with other acid-suppressive therapies. Follow-up remains relatively short, however, and agent-specific long-term estimates are sparse. Gastric candidiasis has also been reported after P-CAB use (Kato et al., 2019). A TriNetX study found that the risk of infectious gastroenteritis within 90 days after initiation of vonoprazan was higher than after initiation of a PPI; this finding suggests a short-term gastrointestinal infection signal but cannot be extrapolated to a long-term causal risk (Al-Fakhouri et al., 2026).

### 5.3 Microscopic colitis and hemorrhagic enterocolitis

An analysis of the Japanese Adverse Drug Event Report (JADER) database identified a disproportionality signal between vonoprazan and microscopic colitis, with time to onset ranging from 7 to 731 days; however, spontaneous reports lack a true exposure denominator and cannot be used to estimate incidence or establish causality (Nakanishi et al., 2025). An earlier JADER study also identified a reporting signal between vonoprazan and hemorrhagic enterocolitis (Kambara et al., 2020). Clinically, acid-suppressive medication history should be reviewed in patients with persistent watery diarrhea, negative routine infectious investigations, or gastrointestinal bleeding.

## 6 Extra-gastric safety signals and drug–drug interactions

### 6.1 Hypomagnesemia

Severe hypomagnesemia has been reported during long-term vonoprazan therapy. Cases associated with prolonged vonoprazan therapy have presented with altered consciousness, seizure-like episodes, hypocalcemia, corrected QT interval (QTc) prolongation, and ventricular premature beats; symptoms and laboratory abnormalities improved after drug discontinuation and magnesium and calcium supplementation (Okamoto et al., 2022; 2026; Aotani et al., 2026). Low urinary magnesium levels and a low fractional excretion of magnesium suggest a mechanism more consistent with impaired intestinal absorption than renal magnesium loss. Among patients with predialysis stage 5 chronic kidney disease, use of PPIs or P-CABs, analyzed as a combined exposure variable, was independently associated with lower serum magnesium levels (Ito et al., 2021). The US prescribing information for vonoprazan advises considering serum magnesium monitoring before treatment and periodically during prolonged therapy, as well as in patients receiving digoxin or medications that can cause hypomagnesemia, such as diuretics (Phathom Pharmaceuticals, 2025).

### 6.2 Hepatic and renal injury and severe cutaneous reactions

A self-controlled case series in patients with cancer showed an increased risk of acute kidney injury during the first 30 days after initiation of a PPI or P-CAB, with no increased risk observed during days 31–365 (Suzuki et al., 2024). Because the two drug classes were analyzed together and anticancer therapy, infection, and dehydration may all have confounded the association, the study does not establish P-CAB-specific nephrotoxicity. In one case, liver injury developed after first exposure to fexuprazan, resolved after withdrawal, and recurred after inadvertent re-exposure 20 months later (Seong et al., 2025). A Korean nationwide claims-based study found a lower risk of coded liver-related outcomes with tegoprazan than with PPIs pooled together, but a higher risk compared with omeprazole alone (Kim et al., 2022). A JADER analysis showed that hepatic and cutaneous events were commonly reported for both PPIs and vonoprazan, although the reporting patterns differed (Kambara et al., 2020). A case of suspected vonoprazan-associated toxic epidermal necrolysis with onset shortly after treatment initiation has also been reported (Pham et al., 2026). These isolated reports warrant clinical awareness but are too sparse to quantify the risk of severe cutaneous reactions during P-CAB therapy.

### 6.3 Drug–drug interactions

Acid-suppressive agents may interact with antiplatelet drugs. Studies in healthy volunteers suggest that vonoprazan can affect platelet-function indices related to clopidogrel or prasugrel, although whether these changes in surrogate endpoints translate into clinical cardiovascular events remains unclear (Kagami et al., 2018). A Korean cohort study comparing P-CABs and PPIs during dual antiplatelet therapy after coronary stent implantation found no significant differences in major adverse cardiovascular composite outcomes or gastrointestinal bleeding (Lee and Je, 2025). Among patients undergoing percutaneous coronary intervention (PCI) and receiving clopidogrel, P2Y12 reaction units and the proportion with high on-treatment platelet reactivity at 3, 6, and 12 months were similar between the tegoprazan and PPI groups (Lee O.-H. et al., 2025). In claims data from a working-age population, lansoprazole users had a higher risk of cardiovascular events than users of vonoprazan and some other PPIs, and the investigators proposed a CYP2C19-related mechanism (Watanabe et al., 2022). However, drug selection, comorbidities, and prescribing indications may have influenced this finding, which should not be interpreted as evidence of a cardioprotective effect of P-CABs.

A crossover study in healthy participants showed that coadministration of vonoprazan increased systemic exposure to atorvastatin and its lactone form and reduced the metabolic ratio of the 2-hydroxy metabolite; the same changes were not observed with tegoprazan (Hwang et al., 2021). The clinical significance of these pharmacokinetic changes and their implications during long-term coadministration remain uncertain.

After kidney transplant recipients were switched from rabeprazole to vonoprazan, the median trough concentration of tacrolimus increased from 5.2 ng/mL to 8.1 ng/mL, and the dose-adjusted concentration also increased significantly; CYP2C19 and CYP3A5 genotypes did not explain the change (Suzuki et al., 2021). Given the narrow therapeutic index of tacrolimus, drug concentrations and renal function should be monitored closely when vonoprazan is initiated, discontinued, or substituted in transplant recipients, and the tacrolimus dose should be adjusted according to monitoring results.

### 6.4 Potential modification of anticancer efficacy and indirect safety signals

Whether gastric acid suppressants affect the efficacy or toxicity of immune checkpoint inhibitors (ICIs) remains uncertain. In a cohort of patients with non-small-cell lung cancer, both PPI and vonoprazan use were associated with shorter progression-free and overall survival, with a stronger association in the vonoprazan group; however, that group included only 22 patients, and disease severity, concomitant medications, and nutritional status may have confounded the results (Takahashi et al., 2025). A multicenter study of patients with hepatocellular carcinoma treated with atezolizumab plus bevacizumab found no association between vonoprazan use and poorer survival outcomes (Takahashi et al., 2026). In patients with small-cell lung cancer receiving carboplatin/etoposide, a retrospective study found that PPI or P-CAB use combined with a low body mass index was associated with febrile neutropenia (Takahashi et al., 2024). A VigiBase analysis identified a disproportionality signal between vonoprazan and cholangitis events among ICI-related reports, although the underlying mechanism remains speculative (Mizuno et al., 2026). Findings across oncology populations are inconsistent and are based largely on small or retrospective cohorts. At present, they are best interpreted as possible drug–treatment interaction signals rather than as evidence of a class-wide effect of P-CABs on anticancer efficacy or toxicity.

## 7 Discussion

The long-term safety profile of P-CABs is best understood according to both the outcome of interest and the type of evidence available. Maintenance trials, particularly those of vonoprazan, provide the strongest evidence for common adverse events, hypergastrinemia, and gastric mucosal changes, whereas rare organ injuries and uncommon extra-gastric events are informed mainly by observational studies, pharmacovigilance analyses, and case reports. Evidence also remains uneven across individual P-CABs because the agents differ in clinical experience, metabolic pathways, and drug–drug interaction profiles. Long-term prescribing should therefore be guided by the continuing indication, treatment intensity, baseline gastric risk, comorbidities, and concomitant medications.

Future research should address several priorities. First, long-term evidence should be extended beyond vonoprazan to tegoprazan, fexuprazan, keverprazan, and zastaprazan, with harmonized definitions of exposure, outcomes, and adverse events. Second, studies of GC should systematically record *H. pylori* status, Operative Link on Gastritis Assessment (OLGA)/Operative Link on Gastric Intestinal Metaplasia Assessment (OLGIM) stage, previous gastric lesions, prior PPI exposure, endoscopic surveillance frequency, and time-varying medication exposure to reduce confounding and reverse causation. Third, longitudinal relationships among gastrin concentrations, histologic changes, endoscopic phenotypes, and hard clinical outcomes should be established, with prespecified procedures for fasting gastrin measurement, biopsy sites, and pathologic interpretation. Fourth, surveillance cohorts for extra-gastric adverse events should incorporate verified prescription exposure, laboratory data, and active event adjudication. Finally, populations from diverse racial and ethnic backgrounds and healthcare systems should be included to avoid uncritical extrapolation of findings from East Asian settings with a high background risk of GC to global populations.

## 8 Conclusion

Long-term safety evidence for P-CABs is derived predominantly from vonoprazan and should not be generalized to the entire class. Hypergastrinemia and adaptive changes in the fundic glands are the most consistently documented long-term effects. The risks of rare or long-latency outcomes remain uncertain. Long-term treatment should therefore be guided by a clear indication, periodic reassessment, the lowest effective dose, and individualized monitoring based on patient risk and agent-specific interactions.
